# Photoinduced electron-transfer chemistry of the bielectrophoric *N*-phthaloyl derivatives of the amino acids tyrosine, histidine and tryptophan

**DOI:** 10.3762/bjoc.7.60

**Published:** 2011-04-26

**Authors:** Axel G Griesbeck, Jörg Neudörfl, Alan de Kiff

**Affiliations:** 1 University of Cologne, Department of Chemistry, Organic Chemistry, Greinstr. 4, D-50939 Köln, Germany; Fax: +49(221)470 5057

**Keywords:** amino acids, decarboxylation, electron transfer, photochemistry, phthalimides

## Abstract

The photochemistry of phthalimide derivatives of the electron-rich amino acids tyrosine, histidine and tryptophan **8**–**10** was studied with respect to photoinduced electron-transfer (PET) induced decarboxylation and Norrish II bond cleavage. Whereas exclusive photodecarboxylation of the tyrosine substrate **8** was observed, the histidine compound **9** resulted in a mixture of histamine and preferential Norrish cleavage. The tryptophan derivative **10** is photochemically inert and shows preferential decarboxylation only when induced by intermolecular PET.

## Introduction

Phthalimides are versatile electron acceptors in photoinduced electron-transfer (PET) reactions. N-Alkylated phthalimides typically absorb in the 295 nm region with extinction coefficients around 10^3^. The quantum yields for intersystem crossing Ф_ISC_ significantly change with the substitution on the imide nitrogen, e.g., Ф_ISC_ = 0.5 for *N*-isobutylphthalimide and Ф_ISC_ < 0.01 for *N*-arylphthalimides [1]. If necessary, the population of the triplet state is also possible by sensitization, e.g., with triplet sensitizers such as acetone or benzophenone. With a triplet energy E_T_ of 293–300 kJ mol^−1^ and a ground-state reduction potential E^0^ of −1.85 V vs Fc/Fc^+^, electronically excited phthalimides are potent electron acceptors [2]. The rich photochemistry of this chromophore has recently been reviewed [3–4]. Intramolecular hydrogen abstraction is an archetype process for electronically excited carbonyl groups (Norrish type II reaction). The 1,4-biradicals formed by γ-CH transfer can undergo several subsequent reactions, among which are secondary H transfer, cyclization, or fragmentation. The excited imido group is at the same time an efficient electron acceptor and can be reduced by numerous donor groups.

**Scheme 1:**
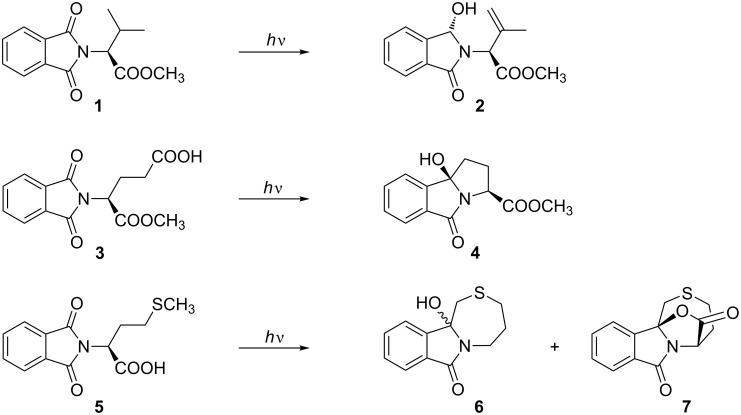
Three phthalimide/amino acid model reactions: Norrish II process of **1**, PET decarboxylation of **3**, PET competition of **5**.

As a model compound for neutral aliphatic amino acids, the photophysical and photochemical properties of *N*-phthaloylvaline methyl ester (**1**) have been studied by nanosecond laser flash photolysis (λ_exc_ = 248 or 308 nm) [5]. The quantum yield of fluorescence is low (Ф_F_ = 10^−2^), whereas that of phosphorescence at −196 °C is large (0.5). The triplet properties of **1** at room temperature and in ethanol at low temperatures are known: Triplet acetone, acetophenone and xanthone in acetonitrile are quenched by **1** via energy transfer; the rate constant is almost diffusion-controlled and somewhat smaller for benzophenone. The sole product from the photolysis of **1** is the double hydrogen transfer product **2**. On the other hand, phthaloyl derivatives of C-unprotected α-amino acids (e.g., derivatives of Gly, Ala, Val, Ile, Phe) undergo efficient photodecarboxylation to yield the corresponding amines, β-amino acids are converted to benzazepines, and γ-amino acids to benzopyrrolizidines (Scheme 1). For example, the glutamic acid derivative **3** resulted in the formation of a diastereoisomeric mixture of benzopyrrolizidinones **4** [6]. For a specific system, *N*-phthaloyl methionine (**5**), we have detected bielectrophoric behavior in that the electron transfer from the thioether group competes efficiently with decarboxylation when the reaction is triplet-sensitized [7]. Photodecarboxylation is followed by PET cyclization to give **6**, whereas the lactone **7** originates from PET-induced sulfur oxidation and radical ion trapping without subsequent decarboxylation. Similar effects were observed for the cysteine and *S*-methyl cysteine derivatives [8].

Other proteinogenic amino acids that, in principle, should also be able to show bielectrophoric behavior with aromatic side chains similar to phenylalanine are tyrosine, histidine and tryptophan. The photochemistry of the phthalimide derivatives of these three amino acids are described in this publication.

## Results

### Synthesis of phthalimide substrates

**Figure 1:**
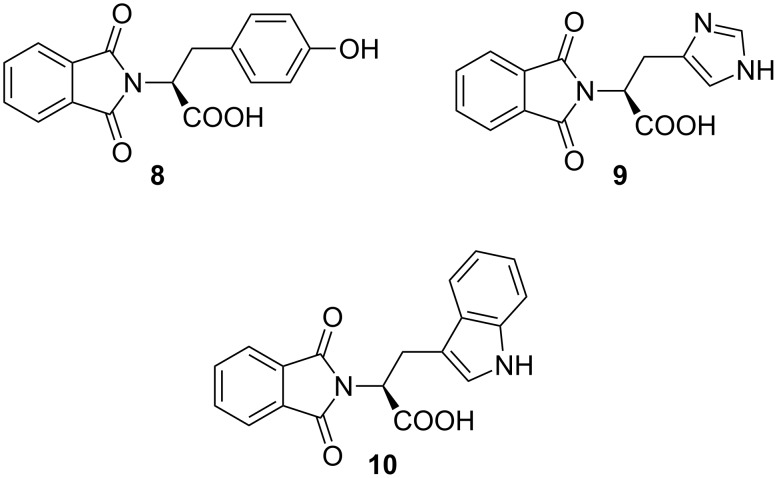
Phthalimides from tyrosine **8**, histidine **9** and tryptophan **10**.

The C-unprotected *N*-phthaloyl amino acids **8**–**10** were available from tyrosine, histidine, and tryptophan (Figure 1). The phthalimide derivatives were prepared either by thermal reaction of phthalic anhydride with the corresponding amino acids or by the Nefkens procedure [9]. The latter procedure yields enantiomerically pure products (by optical rotation), whereas the thermal method leads to partial epimerization.

### Photochemistry of the tyrosine and histidine derivatives **8** and **9**

**Scheme 2:**
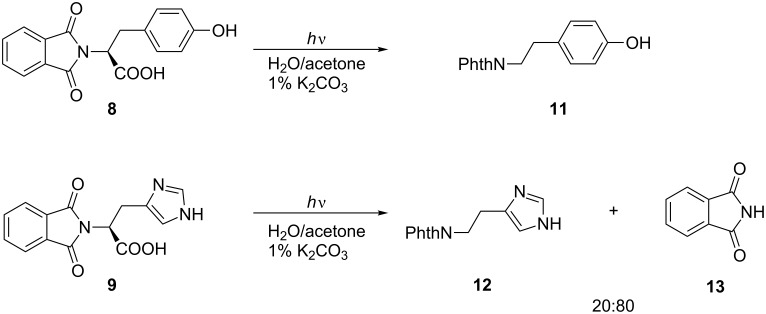
PET decarboxylation/photocleavage of **8** and **9**.

The colorless phthalimides from tyrosine and histidine **8** and **9**, respectively, were photochemically active and gave decarboxylation and cleavage products. In contrast to photolysis in pure acetone [10], irradiation of the tyrosine substrate **8** in basic water/acetone resulted solely in the decarboxylation product **11**. Under identical conditions, the histidine derivative **9** resulted in a 1:4 mixture of phthaloyl histamine **12** and phthalimide (**13**) (Scheme 2). In both cases, the photolyses were clean processes and the products were isolated in high yields after complete conversion. The tyramine derivative **11** was isolated by crystallization in near quantitative yield.

### Photochemistry of the tryptophan derivative **10**

**Figure 2:**
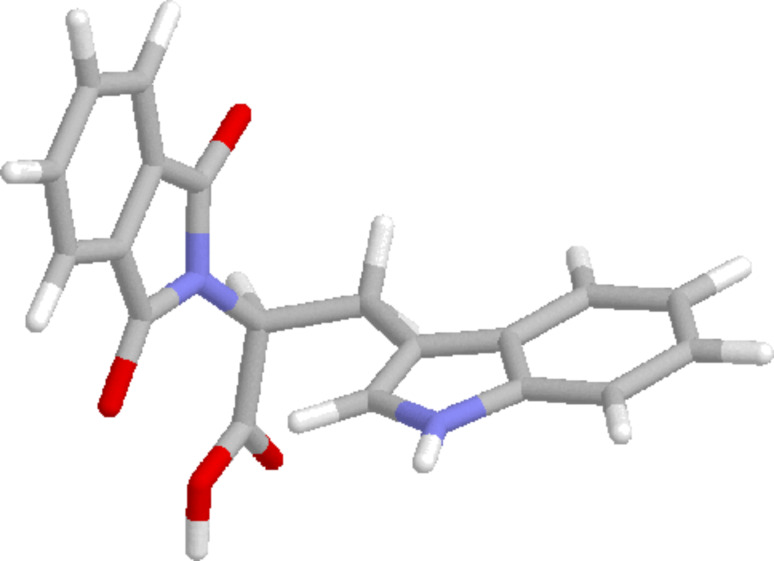
Structure of the tryptophan derivative **10** in the crystal.

**Figure 3:**
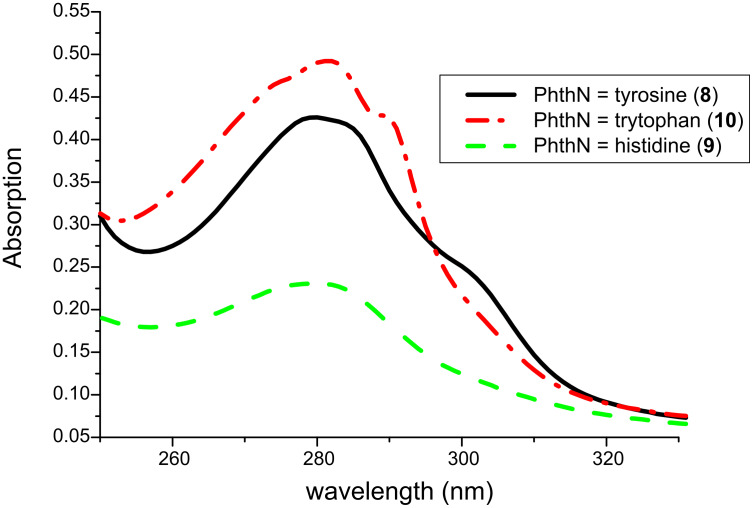
UV–vis absorption spectra of compounds **8**–**10** (*c* = 2 × 10^−4^ in CH_3_OH).

**Scheme 3:**
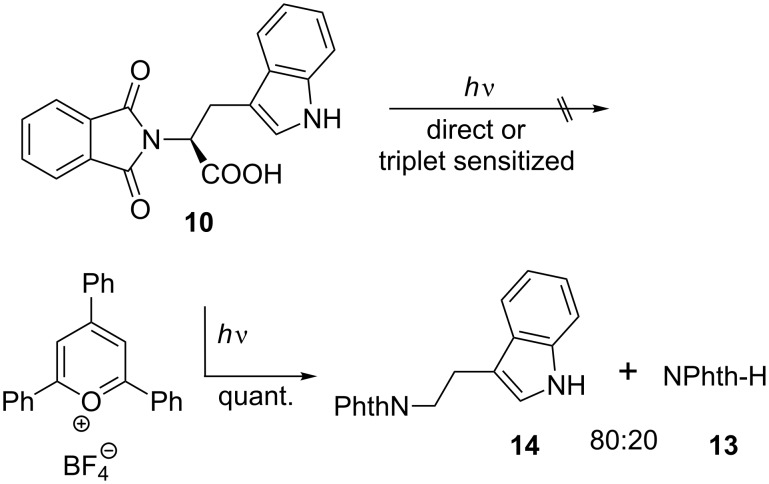
Direct, triplet-sensitized and ET-sensitized photochemistry of **10**.

In contrast to all other – colorless – phthalimides of the proteinogenic amino acid series, the tryptophan derivative **10** is bright yellow. This compound, which we crystallized and whose solid-state structure was determined by X-ray diffraction analysis (Figure 2) [11], has also been described in literature as a yellow compound [12–13]. The color is not the consequence of any impurity and did not vanish after several recrystallizations and, more importantly, was also observed for the methyl ester and the decarboxylation product, the *N*-phthaloyl tryptamine (**14**). Compound **10** represents a donor–*ethylene bridge*–acceptor situation and intramolecular electron transition (ET) is feasible. The UV spectra of the three substrates **8**–**10** (Figure 3) showed in fact two major absorption peaks for **8** and **10** at 280 and 300 nm, whereas the histidine derivative **9** only exhibits the typical phthalimide absorption at λ_max_ = 282 nm. The red-shifted absorption of **10** has a tailing that explains the color of this compound and derivatives as CT state absorption and is additionally red-shifted by 10 nm on changing the solvent from ether to acetonitrile or methanol, respectively. It is remarkable that under solid-state conditions the molecular conformation of **10** in the crystal lattice is synclinal with respect to the indole and the phthalimide groups as well as for the indole and the carboxy groups, indicating a ground-state electronic interaction between the donor indole and the acceptor phthalimide (Figure 2). Such interactions also in solution phase would facilitate decay processes from the excited singlet or triplet state. This concept has also been described by Gawronski et al. from exciton CD Cotton effects that have been measured for a series of bichromophoric systems based on phthalimide–linker–donor triades [12]. Concerning the photochemistry of **10**, a remarkable difference to all other phthalimide derivatives occurred: Neither direct excitation nor triplet (acetone, benzophenone) sensitized conditions led to substrate conversion even after prolonged irradiation. Only the use of the PET catalyst 2,4,6-triphenylpyrylium tetrafluoroborate enabled quantitative conversion to give a 4:1 mixture of the decarboxylation product, *N*-phthaloyl typtamine (**14**) and the cleavage product **13** (Scheme 3).

**Figure 4:**
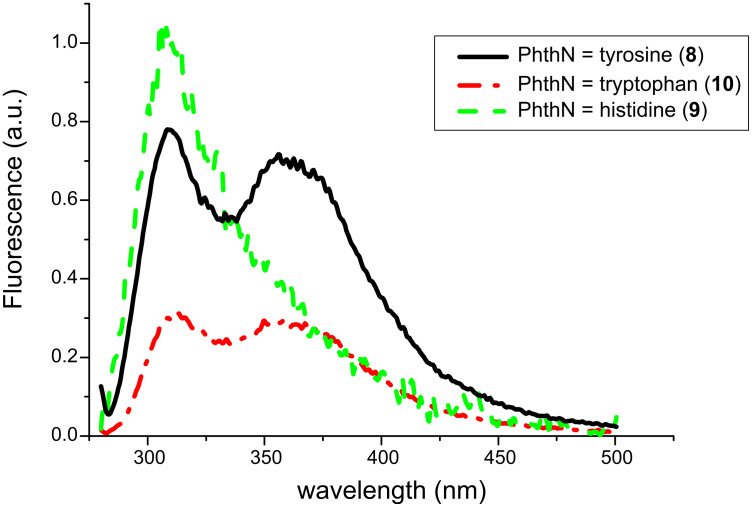
Fluorescence spectra of compounds **8**–**10** (*c* = 6 × 10^−5^ in CH_3_OH).

Additionally, the fluorescence spectra (Figure 4) indicated a charge-transfer excited-state formation from the tyrosine and tryptophan derivatives, **8** and **10**, respectively, by showing dual emission at 310 and 370 nm.

## Discussion

Two reaction modes were observed with the phthalimides **8**–**10**: (a) Norrish type II cleavage of the central N–C bond to give the N-unsubstituted phthalimide **13**, and (b) photochemical decarboxylation. Analysis of the energetics of the PET between the phthalimide acceptor (see Introduction) and the arene donor side chains of the corresponding amino acid leads to the conclusion that PET is exergonic with Δ*G* = −0.1 to −0.3 V for all cases: The redox potentials of the three amino acids tryptophan, histidine and tyrosine are reported to be 1.02 V [14], 1.17 V [15] and 0.93 V [16].

**Scheme 4:**
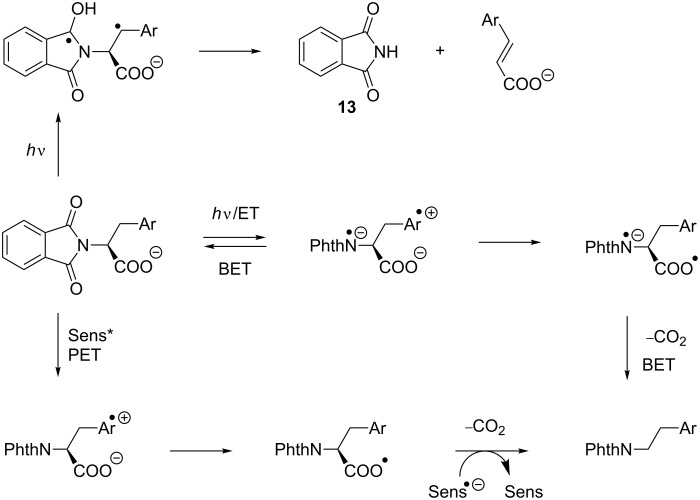
Mechanistic scenario.

Due to this narrow redox potential window, similar electron-transfer properties can be expected. Among the amino acids investigated herein, tryptophan is described in biological electron-transfer processes as the most active hole transport molecule [17]. Thus, it can be concluded that the excited charge-transfer state from **10**, as can be interpreted from its fluorescence spectrum, is rapidly deactivated radiatively as well as non-radiatively by back electron transfer (BET). In contrast to **10**, electron transfer from the electronically excited tyrosine compound **8** is followed by oxidation of the carboxyl anion and subsequent decarboxylation to give *N*-phthaloyl tyramine (**11**). If BET from the radical cation state of **10** can be retarded (i.e., produced by an intermolecular PET), the corresponding decarboxylation proceeds to give *N*-phthaloyl tryptamine (**14**) (Scheme 3). The histidine derivative **9** is expected to undergo PET with oxidation of the aryl group with the lowest efficiency and thus a Norrish II process (γ-hydrogen transfer with subsequent central bond cleavage) prevails here (Scheme 4). This process, which was also detected as the minor pathway for the histidine case can, of course, also be described as an electron-transfer induced process followed by α-deprotonation.

## Conclusion

The photochemistry of three bielectrophoric phthaloyl derivatives of tyrosine (**8**), histidine (**9**) and tryptophan (**10**) follows two distinct pathways, fragmentation and decarboxylation. Both routes can be described as initiated by electron transfer. Intramolecular transfer in the tyrosine substrate **8** leads to efficient decarboxylation whereas for the tryptophan substrate **10** this process can only be initiated by intermolecular electron transfer suggesting efficient back electron transfer in the photoexcited substrate.

## Experimental

**Synthesis of *N*-phthaloyl tyrosine (8)** [18]. A well ground mixture of 2.00 g (13 mmol) of L-tyrosine and 2.45 g (13 mmol) of phthalic anhydride were added to a 10 mL flask with a magnetic stirring bar. The flask was heated in an oil bath, stirred at 150–160 °C for 20 min and then the mixture was allowed to cool to room temperature. Recrystallization of the crude solid material from ethanol gave 3.67 g of *N*-phthaloyl tyrosine (**8**) (88%) as colorless needles; mp 161–163 °C (lit. 162–164 °C); [α]_D_^20^ −183.8 (*c* 1.0, EtOH) (lit. −182.4); ^1^H NMR (300 MHz, DMSO-*d*_6_): δ (ppm) = 3.22 (dd, 2H, *J*_1_ = 13.6 Hz, *J*_2_ = 11.8 Hz), 5.01 (dd, 1H, *J*_1_ = 11.9 Hz, *J*_2_ = 5.0 Hz), 6.53 (d, 1H, *J* = 8.5 Hz), 6.91 (d, *J* = 8.5 Hz), 7.84 (s, 4H), 9.14 (s, COOH). ^13^C NMR (75 MHz, DMSO-*d*_6_): δ (ppm) = 33.1 (t, 1C), 53.2 (s, 1C), 115.1 (d, 2C), 123.4 (d, 2C), 127.2 (s, 1C), 129.7 (d, 2C), 130.8 (s, 2C), 134.9 (d, 2C), 155.8 (s, 1C), 167.1 (s, 2C), 170.2 (s, 1C).

**Synthesis of *N*-phthaloyl histidine (9)** [19]. L-histidine 3.0 g (14 mmol) and 1.52 g (14 mmol) of Na_2_CO_3_ were dissolved in 100 mL water and 3.14 g (14 mmol) of *N*-carbethoxy phthalimide was added to the solution. The mixture was allowed to stir at room temperature for 2 h. After acidification with 2 M HCl, the solvent was removed under reduced pressure. The colorless residue was heated under reflux in 10 mL MeOH for 20 min. After cooling, the residue was removed by filtration and washed with 100 mL MeOH to give 3.40 g of *N*-phthaloyl histidine (**9**) (83%) as a colorless solid; mp >260 °C; ^1^H NMR (300 MHz, DMSO-*d*_6_): δ (ppm) = 3.37 (d, 2H, *J* = 6.6 Hz, H 7), 4.91 (dd, 1H, *J*_1_ = 9.5 Hz, *J*_2_ = 6.5 Hz), 6.81 (s, 1H), 7.73 (s, 1H), 7.84 (s, 4H). ^13^C NMR (75 MHz, DMSO-*d*_6_): δ (ppm) = 33.1 (t, 1C), 53.2 (s, 1C), 115.1 (d, 2C), 123.4 (d, 2C), 127.2 (s, 1C), 129.7 (d, 2C), 130.8 (s, 2C), 134.9 (d, 2C), 155.8 (s, 1C), 167.1 (s, 2C), 170.2 (s, 1C).

**Synthesis of *N*-phthaloyl tryptophan (10)** [18]. A mixture of 2 g (9.8 mmol) of L-tryptophan and 1.04 g (9.8 mmol) of Na_2_CO_3_ was dissolved in 100 mL of water. To this solution, 2.15 g (9.8 mmol) of *N*-carbethoxy phthalimide were added. The mixture was stirred at room temperature for 1 h. After filtration the solution was acidified with 2 M HCl and the precipitate collected. Recrystallization from aqueous acetone gave 3.12 g of *N*-phthaloyl tryptophan (**10**) (95%) as yellow needles; mp 170 °C; [α]_D_^20^ −247.5 (*c* 1.0, EtOH) (lit: −249.6); ^1^H NMR (300 MHz, DMSO-*d*_6_): δ (ppm) = 3.55–3.62 (m, 2H), 5.13 (dd, 1H, *J*_1_ = 10 Hz, *J*_2_ = 6.7 Hz), 6.89 (t, 1H, *J* = 7.3 Hz), 7.00 (t, 1H, *J* = 8 Hz), 7.49 (d, 1H, *J* = 8.1 Hz), 7.26 (d, 1H, *J* = 8.2 Hz), 7.80 (s, 4H), 10.74 (s, COOH). ^13^C NMR (75 MHz, DMSO-*d*_6_): δ (ppm) = 24.1 (t, 1C), 52.7 (d, 1C), 109.8 (s, 1C), 111.5 (d, 1C), 117.9 (d, 1C), 118.5 (d, 1C), 121.0 (d, 1C), 123.4 (d, 2C), 126.9 (s, 1C), 130.9 (s, 2C), 134.9 (d, 2C), 136.1 (s, 1C), 167.2 (s, 2C), 170.4 (s, 1C).

**Photolysis of *N*-phthaloyl tyrosine (8)**. A water-cooled solution (*c* = 2.1 × 10^−3^ mol/l) of **8** and 0.5 equiv K_2_CO_3_ in 100 mL of an acetone/water mixture (1:1) was irradiated at 300 nm (lamps: 8 × 3000 Å, 800 W, 300 ± 10 nm) for 12 h under a nitrogen atmosphere. The product was removed by filtration and washed with water to give 396 mg of *N*-phthaloyl tyramine (**11**) (95%) as a colorless solid; mp 171–174 °C; ^1^H NMR (300 MHz, DMSO-*d*_6_): δ (ppm) = 2.77 (t, 2H, *J* = 7.5 Hz), 3.73 (t, 2H, *J* = 7.5 Hz), 6.59 (d, 2H, *J* = 8.5 Hz), 6.92 (d, 2H, *J* = 8.5 Hz), 7.82 (s, 2H), 7.83 (s, 2H). ^13^C NMR (75 MHz, DMSO-*d*_6_): δ (ppm) = 32.9 (t, 1C), 40.3 (t, 1C), 115.5 (d, 2C), 123.0 (d, 2C), 127.0 (s, 1C), 129.4 (d, 2C, C8/C8’), 131.5 (s, 2C), 134.4 (d, 2C), 157.2 (s, 1C), 167.7 (s, 2C).

**Photolysis of* N*-phthaloyl histidine (9)**. A water-cooled solution (*c* = 2.1 × 10^−3^ mol/l) of **9** and 0.5 equiv K_2_CO_3_ in 100 mL of an acetone/water mixture (1:1) was irradiated at 300 nm (lamps: 8 × 3000 Å, 800 W, 300 ± 10 nm) under a nitrogen atmosphere. After 24 h the solution was acidified with 2 M HCl and the solvent was removed under reduced pressure. The product composition [*N*-phthaloyl histamine (**12**)/phthalimide (**13**)] was determined by ^1^H NMR spectroscopy.

**12:** 20% (by ^1^H NMR); mp 173–175 °C; ^1^H NMR (300 MHz, DMSO-*d*_6_): δ (ppm) = 2.91 (t, 2H, *J* = 6.6 Hz), 3.88 (t, 2H, *J* = 6.6 Hz), 6.79 (s, 1H), 7.70 (s, 1H), 7.80 (s, 4H). ^13^C NMR (75 MHz, DMSO-*d*_6_): δ (ppm) = 24.0 (t, 1C), 37.9 (d, 1C, C5), 116.1 (d, 1C), 123.1 (d, 2C), 131.1 (s, 2C), 132.9 (d, 1C), 134.5 (d, 2C), 138.4 (s, 1C), 167.9 (s, 2C, C4/C4’).

**13:** 80% (by ^1^H NMR); ^1^H NMR (300 MHz, DMSO-*d*_6_): δ (ppm) = 7.75 (s, 4H). ^13^C NMR (75 MHz, DMSO-*d*_6_): δ (ppm) = 123.0 (d, 2C), 132.1 (s, 2C), 134.7 (d, 2C), 167.2 (s, 2C).

**Photolysis of* N*-phthaloyl tryptophan (10)**. A 100 mL solution (*c* = 0.01 mol/l) of **10** and 5.4 × 10^−3^ mmol 2,4,6-triphenylpyrylium tetrafluoroborate in acetonitrile was irradiated at 350 nm (RPR-3500 Å lamp: 6 × 3500 Å, 400 W, 350 ± 25 nm) for 4 d under a nitrogen atmosphere. After removal of the solvent under reduced pressure, the ratio of products was determined by ^1^H NMR spectroscopy. Silica gel flash chromatography (cyclohexane/ethylacetate) of the residue afforded *N*-phthaloyl tryptamine (**14**) as a yellow solid and phthalimide (**13**) as a colorless solid.

**14:** 80% (by ^1^H NMR); mp 173–175 °C; ^1^H NMR (300 MHz, acetone-*d*_6_): δ (ppm) = 3.13 (t, 2H, *J* = 7.7 Hz), 3.95 (t, 2H, *J* = 7.7 Hz), 7.01 (t, 1H, *J* = 7.4 Hz), 7.09 (t, 1H, *J* = 7.8 Hz), 7.20 (s, 1H), 7.37 (d, 1H, *J* = 7.9 Hz), 7.67 (d, 1H, *J* = 87.7 Hz), 7.81 (s, 4H). ^13^C NMR (75 MHz, acetone-*d*_6_): δ (ppm) = 24.2 (t, 1C), 38.4 (t, 1C), 111.4 (d, 1C), 118.3 (d, 1C), 118.7 (d, 1C), 121.3 (d, 1C), 122.9 (d, 2C), 127.6 (s, 1C), 132.3 (s, 2C), 134.1 (d, 2C), 135.9 (s, 1C), 167.9 (s, 2C).
